# Proposal of Classification for Management of Molar Extraction Sockets and Decision Making for Immediate Implant Placement: A Technical Report

**DOI:** 10.7759/cureus.93968

**Published:** 2025-10-06

**Authors:** Mehdi Ekhlasmandkermani, Fatemeh Goudarzimoghaddam, Emir Ilkerli

**Affiliations:** 1 Department of Periodontics, Kerman University of Medical Sciences, Kerman, IRN; 2 Department of Periodontics, School of Dentistry, Babol University of Medical Sciences, Babol, IRN; 3 Department of Orthopedics and Traumatology, OsteMed Klinik Bremervörde, Bremervörde, DEU

**Keywords:** classification, dental implants, immediate implants, tooth extraction, tooth socket

## Abstract

In cases where adequate primary stability (PS) is established, the immediate implant placement (IIP) in molar sockets is considered a reliable treatment option that significantly reduces the overall treatment time. Several key factors contribute to the success of IIP in molar sockets, notably the presence of sufficient inter-radicular septa, which is essential for achieving the necessary PS in cases of elongated roots and also preventing buccal plate collapse. Furthermore, the properties of the buccal bone plate (BBP) may have a significant impact on the final morphology of the ridge following the healing period. This technical note aims to offer a classification system for multi-rooted molar sockets, along with treatment guidelines that consider crucial variables such as morphology, biological factors and the mechanical properties of the socket. The prevalent classification of molar sockets documented in the literature is based on the inter-radicular septum. This study introduces a different classification along with treatment recommendations aimed at enhancing the management of multi-rooted molar sockets during IIP. The proposed classification focuses on two critical variables: the presence of inter-radicular septa and BBP, to achieve optimal PS. According to the different conditions of the septal bone and buccal bone plate, the molar socket categorized into four distinct types - Type 1: Intact bony walls with intact septal bone; Type 2: Compromised bony walls while the septal bone remains intact; Type 3: Intact bony walls with a compromised septal bone; Type 4: Both bony walls and septal bone are compromised. This classification is crucial for understanding the structural integrity of molar sockets and guiding appropriate clinical interventions. This biomechanical classification emphasizes the beneficial role of the septal bone, along with the situations of the BBT, in preserving the vascular supply to the buccal plate, thereby reducing the risk of its collapse. This classification serves as a valuable framework for managing sockets during IIP. In all socket types, achieving PS is paramount for successful implant placement procedures.

## Introduction

Following a tooth extraction, practitioners typically consider two primary treatment options: Immediate implant placement (IIP) or alveolar ridge preservation (ARP) [1]. It is worth noting that there is some debate in the literature regarding the efficacy of ARP. Therefore, allowing the socket to undergo natural healing may also be viable when dealing with a fresh socket [2].

In terms of timing, the technique of placing an implant within a socket immediately following tooth extraction is referred to as IIP. While IIP is often regarded as synonymous with fresh socket implant placement, it is important to note that some literature categorizes implant placements performed up to one week after tooth extraction as falling within the scope of immediate implants [1].

The concept of IIP is well-established in foundational research [3]. However, the variations observed in the morphology of dental sockets present challenges in developing a comprehensive classification system and reproducible guidelines for IIP. Numerous classifications have been proposed for dental sockets, each focusing on one or more variables [4-9]. Most of these classifications pertain primarily to single-rooted sockets, with the buccal bone plate identified as the main variable due to its susceptibility to resorption compared to other walls. Following tooth extraction and the subsequent interruption of blood supply through the periodontal ligament (PDL), the bundle bone is significantly affected, leading to its resorption. Consequently, the bundle bone is considered a tooth-dependent structure [10].

Adequate primary stability (PS) is essential for IIP [3]. In the molar regions, the inter-radicular septum plays a crucial role in achieving PS in IIP. The connection between the septal bone and the buccal bone plate (BBP), examined from both biological and mechanical perspectives, plays a significant role in influencing socket changes during IIP [11]. This article introduces a biomechanical classification for multi-root sockets, focusing on the two primary components: the septal bone and the BBP.

In cases where the patient is systemically compromised and there is uncertainty regarding their immune response and metabolic function, it is advisable to postpone implant placement immediately following extraction. This approach facilitates careful observation of the metabolic process within the socket [12].

## Technical report

**Figure 1:**
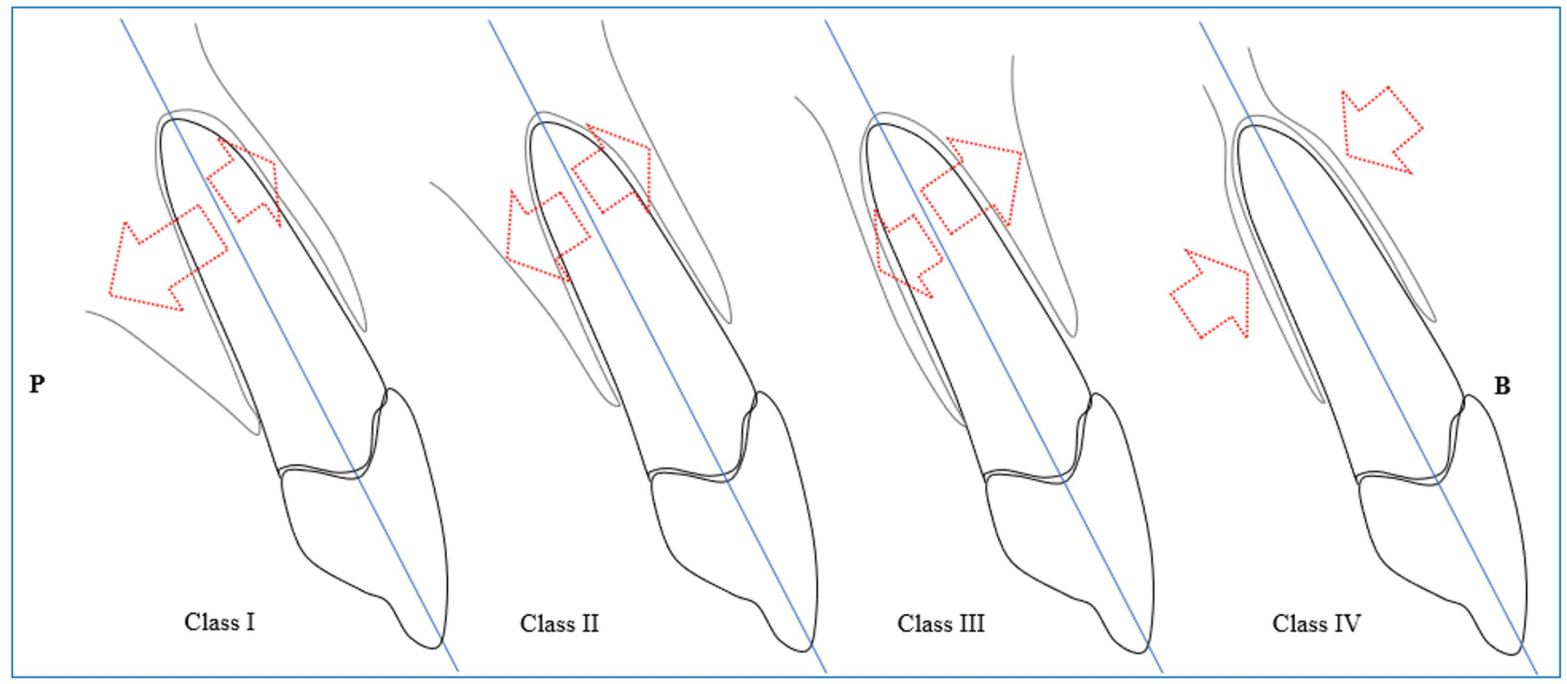
The placement of the roots according to the sagittal root position (SRP) classification (Author's own creation). It is important to note the variations in the size of the arrows, as they indicate the distance from the plate.

**Figure 2:**
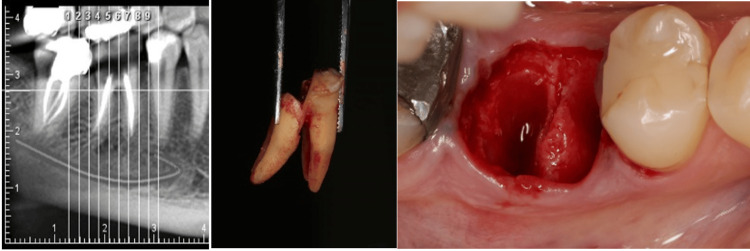
Diverging roots. The starting point of the septal bone in the coronal socket and the degree of divergence of the molar tooth roots affect the decision to place the implant immediately.

**Figure 3:**
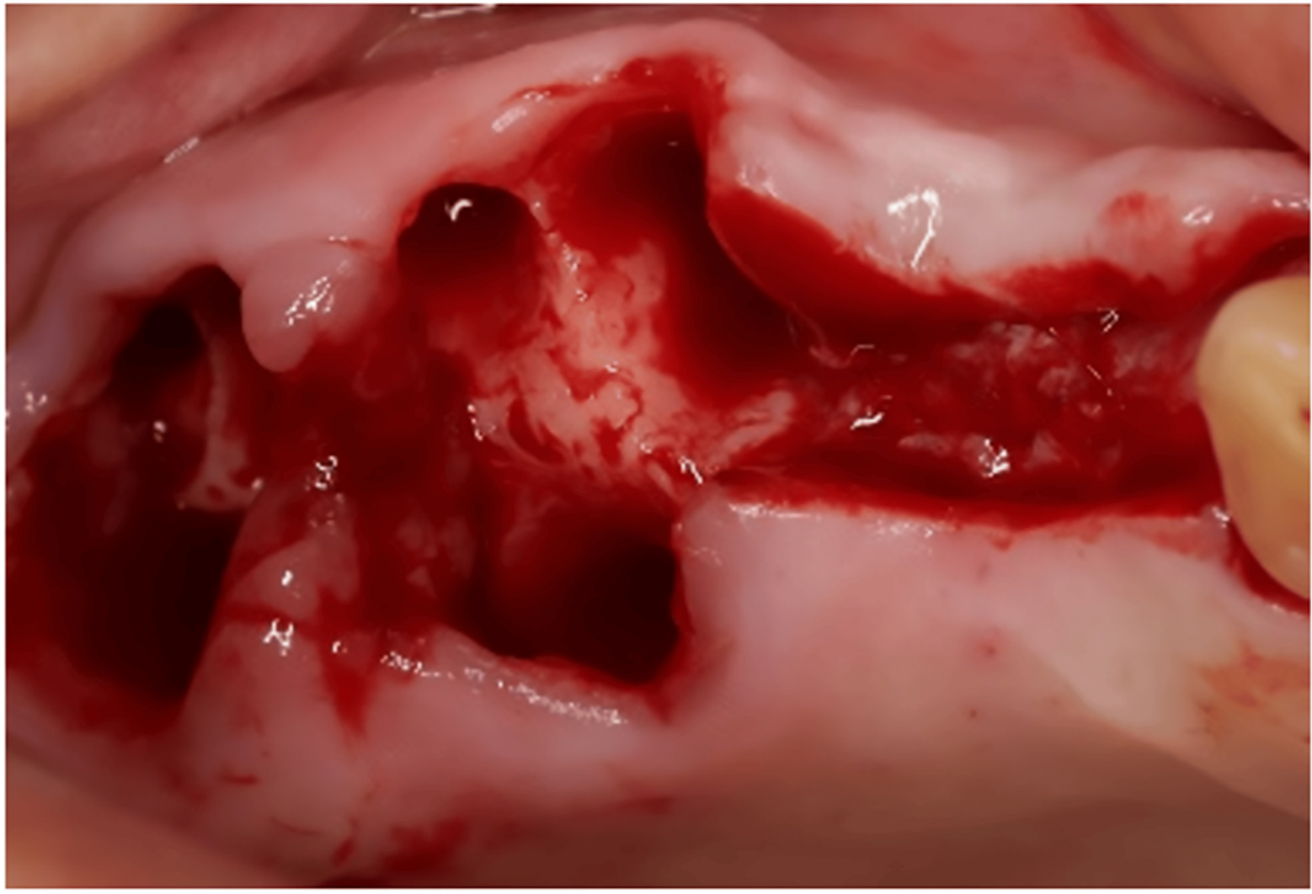
Septal bone inclination. In the maxillary three-rooted molar, if the septal bone is intact, a part of it extends from the center to the buccal side and connects to this wall.

Adequate PS is essential for IIP [3]. In cases involving single-rooted sockets, this stability is typically achieved through the presence of remaining bone on the palatal or lingual side. According to the sagittal root position (SRP) classification, PS can be attained in class 1 [13]. Figure 1 presents a schematic illustration of this classification. In the molar regions, the inter-radicular septum plays a crucial role in achieving PS in IIP. The angle of root placement and the starting position of the furcation within the socket can significantly influence the dimensions of the inter-radicular septum. It is noteworthy that converging roots offer an optimal configuration for utilizing the septal bone (Figure 2) [14]. The anatomy of multi-rooted molar teeth exhibits notable differences between the mandible and maxilla; however, both regions share a common characteristic in that the inter-radicular septum demonstrates a buccal inclination. In the mandible, the septal bone is oriented from the lingual aspect towards the buccal side (Figure 2). Conversely, in the maxilla, particularly when the molar teeth possess three roots, a section of the septal bone extends from the central area towards the buccal aspect, positioned between the mesiobuccal and distobuccal roots (Figure 3).

Biomechanical classification types

1) Intact Bony Wall: This term refers to the preservation of the buccal wall's integrity following tooth extraction. 2) Intact Septal Bone: This indicates the presence of a septum with sufficient width to ensure blood supply, connected to the buccal plate coronally enough to serve as a tent. 3) Compromised Bony Wall: This term signifies the existence of any bony dehiscence in the buccal plate after tooth extraction. 4) Compromised Septal Bone: This denotes a condition where the septal bone is either too thin to preserve blood supply adequately or exhibits structural defects in the buccal aspect, impacting its connection to the buccal plate. The deep positioning of the coronal aspect of the septal bone, attributed to the elongated furcation in molar teeth, may pose a challenge to the integrity of the socket and the corono-apical orientation of the implant. This type of socket can be classified as a Compromised Septal Bone.

**Figure 4:**
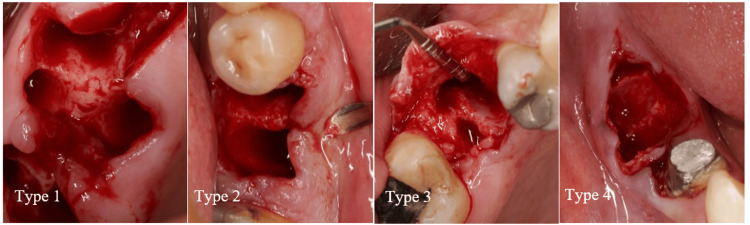
Biomechanical classification. Type 1: Intact bony walls, intact septal bone; Type 2: Compromised bony walls, intact septal bone; Type 3: Intact bony walls, compromised septal bone; Type 4: Compromised bony walls, compromised septal bone.

**Figure 5:**
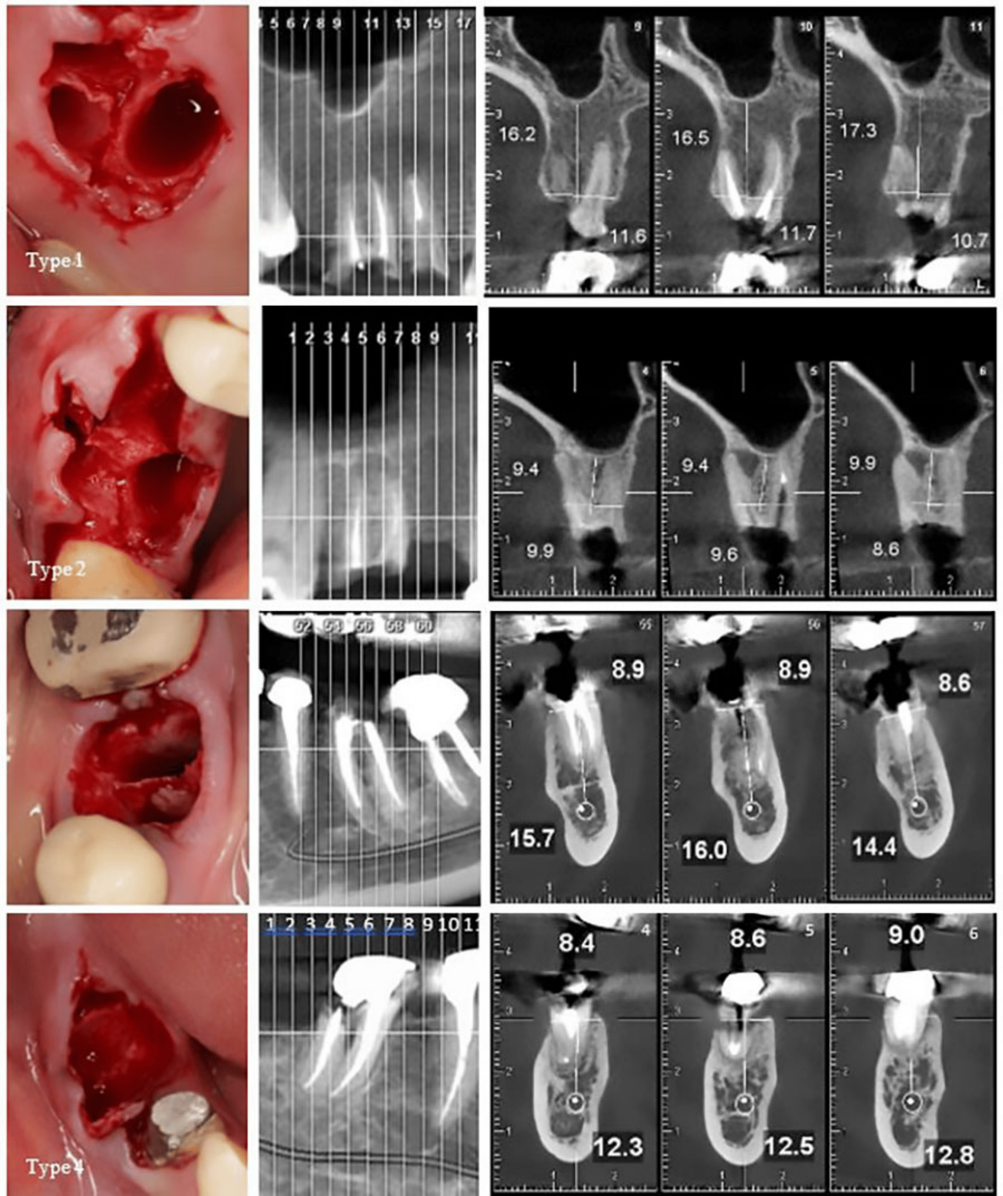
Biomechanical classification based on cone beam computed tomography (CBCT). Type 1. Intact bony wall, intact septal bone: In this type, the divergence of the roots has resulted in the septal bone exhibiting a favorable potential for vascularization to the buccal plate, making it suitable for IP. The CBCT analysis in cut number 10 clearly demonstrates both the substantial width of the septal bone and the integrity of the buccal plate. Type 2. Compromised bony walls, intact septal bone: In this type, despite damage to the buccal plate, the septal bone maintains a strong potential for the IP. When the extension of the septal bone is preserved in proximity to the buccal plate, it plays a crucial role in preventing soft tissue collapse, even in the presence of buccal plate destruction. The CBCT reveals a substantial septal bone situated between the roots. However, it is important to note that in cut number 6, the protrusion of the distal root coincided with the compromise of the buccal plate. Type 3. Intact bony wall, compromised septal bone: In this type, the septal bone is either compromised or lacks the inherent capacity to support an implant successfully, despite the buccal plate maintaining its structural integrity. Consequently, the buccal plate does not contribute to the preservation of the underlying septal bone. The CBCT reveals a convergence of the roots and significant thinning of the septal bone. Furthermore, the sequence of cross-sectional cuts corroborates the diminished thickness of the septal bone. Type 4. Compromised bony wall, compromised septal bone: In this type, the bone resources are compromised both for IP and for maintaining an adequate blood supply. As a result, there is a significantly increased risk of alveolar ridge resorption during the healing phase. The CBCT reveals convergence of the roots and a reduction in the thickness of the septal bone. Furthermore, the presence of furcation involvement observed in the radiograph is indicative of buccal plate destruction, as clearly illustrated in cut number 5.

Through this classification, four distinct types are identified (Figures 4, 5).

Type 1: Intact bony walls, intact septal bone (act as a tent for buccal plate). If PS is possible for IIP, fill the gaps for Bone Bridging [15]. Type 2: Compromised bony walls, intact septal bone (act as a tent for soft tissue). If PS is possible for IIP, fill the gaps with any bone material type, use a barrier membrane and consider the soft tissue for contour augmentation. Type 3: Intact bony walls, compromised septal bone. IIP depends on primary stability. In thin buccal bone, a safe jumping distance is mandatory. Fill the gaps with slow resorption bone material and consider the soft tissue for contour augmentation. In instances involving deep septal bone that result in exposed threads in the coronal portion of implants, it is advisable to achieve optimal sealing over the socket. This can be accomplished through the use of a customized healing abutment or by implementing soft tissue grafting. Type 4: Compromised bony walls, compromised septal bone. IIP depends on primary stability. Fill the gaps, use a barrier membrane, consider the soft tissue for contour augmentation and seal the orifice of the socket.

Table 1 provides an overview of this classification.

**Table 1:** A comprehensive summary of biomechanical classification. IIP: Immediate Implant Placement, BB: Buccal Bone, GBR: Guided Bone Regeneration

Type	Buccal Bone	Septal Bone	Treatment plan in case of enough primary stability
I	Intact	Intact	IIP + Gap filling
II	Compromised	Intact	IIP + GBR
III	Intact	Compromised	In thick BB: IIP + Gap filling / In thin BB: IIP + Soft tissue/Orifice management
IV	Compromised	Compromised	IIP + GBR + Contour management + Socket seal

## Discussion

In 2013, Smith and Tarnow introduced an alternative classification system for immediate implant placement in molar regions. The primary criterion for this classification is the extent to which the surrounding septal bone covers the implant body. In some instances, the width of the septal bone allows for the complete integration of the implant within the bone (class 1). Conversely, in the absence of septal bone coverage, it becomes necessary to utilize other anatomic structures, such as the apical region of the socket, to achieve proper support for PS (class 3) [14].

To maintain the integrity of the septal bone structure, it is advisable to utilize the tooth section for the independent extraction of the roots. Following the removal of the roots, it is crucial to assess the septal bone and BBT if the soft tissue maintains its normal position. In the newly presented classification, the septal bone is considered more influential than the buccal plate. When a wide septal bone is present and connected to the buccal plate, two perspectives on the role of the septum are proposed. From a biological standpoint, the blood supply to the buccal wall may be compromised following tooth extraction. A wide septum, in conjunction with the buccal plate, has the potential to enhance blood supply to this area and improve its regenerative capacity. This phenomenon has been explored and validated, even in single-root sockets, concerning interproximal bone [7]. The second view to consider is the mechanical aspect. The presence of a septum connected to the buccal plate may serve as a barrier to the collapse of this plate during socket changes. Thus, from a biomechanical standpoint, the septal bone plays a significant role in mitigating socket alterations following tooth extraction.

In all outlined types, IIP is applicable only when the PS is attainable. Furthermore, it is essential that the soft tissue has not undergone resorption before tooth extraction.

**Figure 6:**
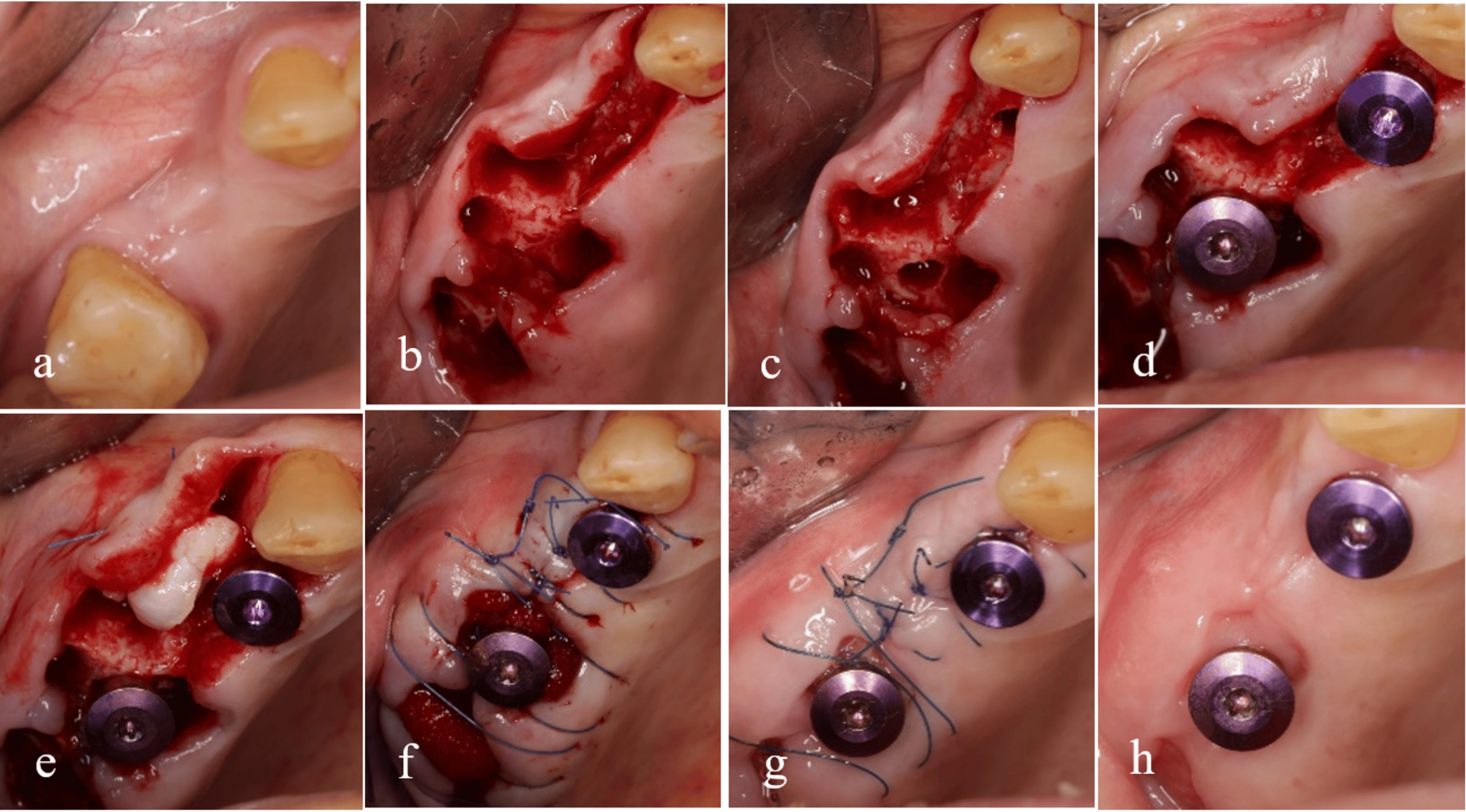
Type 1 of the biomechanical classification. The integrity of the septal bone and bony wall is preserved (a). There is enough bone to completely cover the implant in the septum (b). To correct the adjacent contour, a soft tissue graft prepared from the tuberosity was used (e). The remaining gap was filled without the need for bone material and only with the help of Gelfoam (f). Two and four weeks later, the condition of the soft tissue has stabilized (g, h).

Biomechanical classification has four types:

In Type 1 biomechanical classification, both biological and mechanical aspects are considered in relation to the septum. In this classification, the BBT is not deemed significant for the survival rate of the implant. The primary role of BBT in this category pertains to its impacts on the buccal ridge contour, which can be effectively addressed through soft tissue grafting. An illustrative example of this type, particularly in the scenario of IIP, is presented in Figure 6.

**Figure 7:**
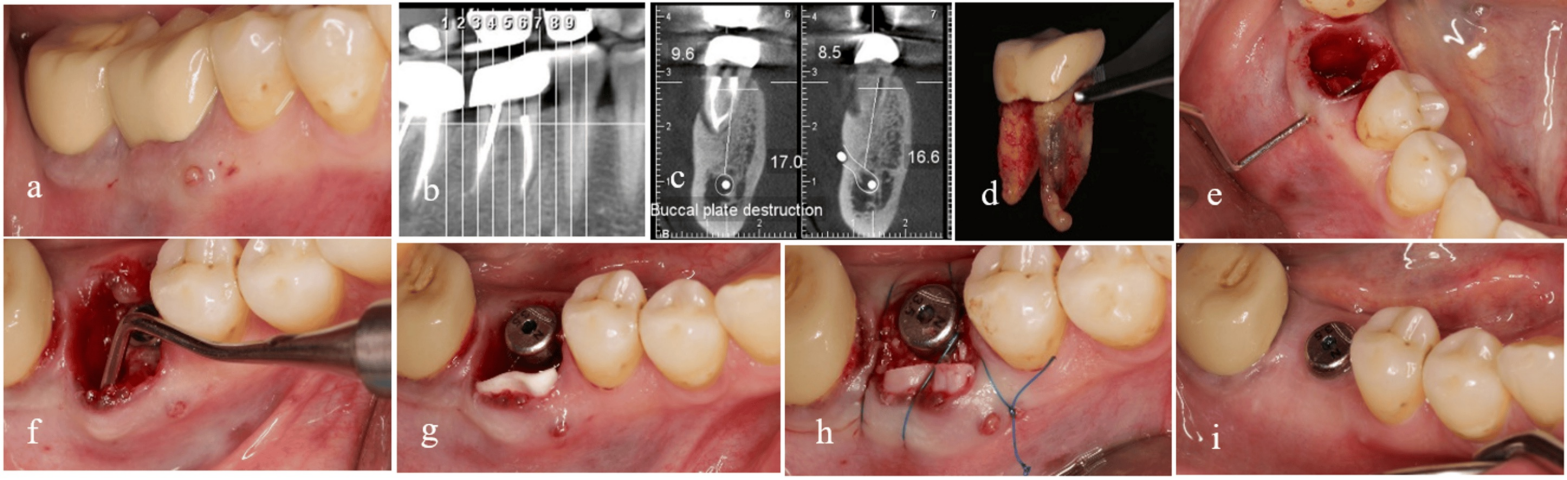
Type 2 of the biomechanical classification. Buccal plate integrity is lost due to vertical root fracture and abscess formation, but an adequate septum is present for immediate implant placement (a-e). After placing the implant, a blunt tool such as a tunneling tool is used to separate the periosteum from the remaining buccal bone (f). A barrier membrane is placed between the periosteum and the remaining buccal bone (g). A slow-absorbing bone material is used to fill the gap under the membrane and sutures are used to preserve the clot inside the bone material (h). The final image, just 10 days after immediate implant placement, shows coronal flooding by soft tissue (i).

In type 2, the buccal plate is destroyed; however, there is still adequate septal bone to play a mechanical tenting role, minimizing soft tissue collapse. To mitigate the internal encroachment of soft tissue, it is recommended to utilize a barrier membrane at the site of the compromised plate. Additionally, filling the internal gap with slow-resorption bone material can further reduce soft tissue collapse. Figure 7 illustrates the management of the socket in type 2. In this particular instance, a vertical root fracture led to the formation of an abscess and the destruction of the buccal plate.

In type 3, where the septal bone is either damaged, in a deep position or demonstrates inadequate regenerative potential, its tenting role in providing structural support is compromised; however, the buccal plate remains intact. The BBT will influence the subsequent decision-making process in this category. If the BBT is more than 1 mm, the changes within the socket will be minimized. Consequently, if sufficient primary stability for the implant is attained, utilizing any bone material to fill the gap will be appropriate to facilitate bone bridging. Conversely, if the buccal plate is categorized as thin (1 mm or less), the use of slow-resorbing bone material is recommended. In such cases, soft tissue grafting may also be indicated to mitigate the potential collapse of the buccal contour. In instances where a deep septal bone leads to exposed threads in the coronal portion of the implants, it is advisable to ensure comprehensive sealing over the socket to prevent complications. This can be achieved through the use of either a customized healing abutment or soft tissue grafting.

In this particular type of socket, it is critical to recognize that when the buccal plate is thin yet retains its structural integrity, the removal of the periosteum from the buccal plate for the placement of a barrier membrane is not aligned with the principles of guided bone regeneration (GBR). This approach aims to minimize buccal plate collapse. It is essential to note that this intervention does not positively contribute to the clinical outcomes associated with IIP; instead, it may lead to an increase in buccal plate resorption [16].

**Figure 8:**
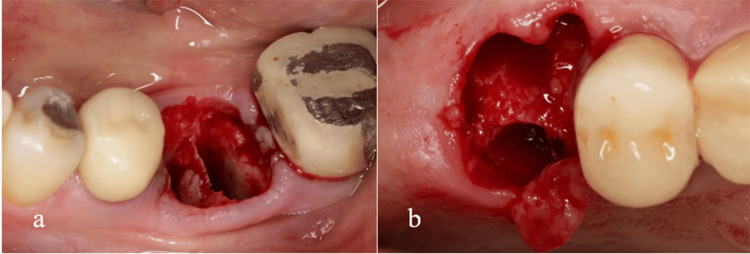
Type 3 of the biomechanical classification. Two types of socket subtype 3 with compromised septal bone. Mandibular first molar with very thin septum (a). Maxillary first molar with destruction of the buccal part of the septum (b). In both cases, buccal bone thickness (BBT) will affect socket changes during the restoration process.

Figure 8 illustrates two distinct conditions of compromised septal bone. In the first scenario, the buccal portion of the septum is compromised, yet the remaining structure is sufficiently robust to support the placement of an implant. In the second scenario, while the integrity of the septal bone is maintained, its diminished thickness renders it inadequate for providing vascular support to the buccal wall.

**Figure 9:**
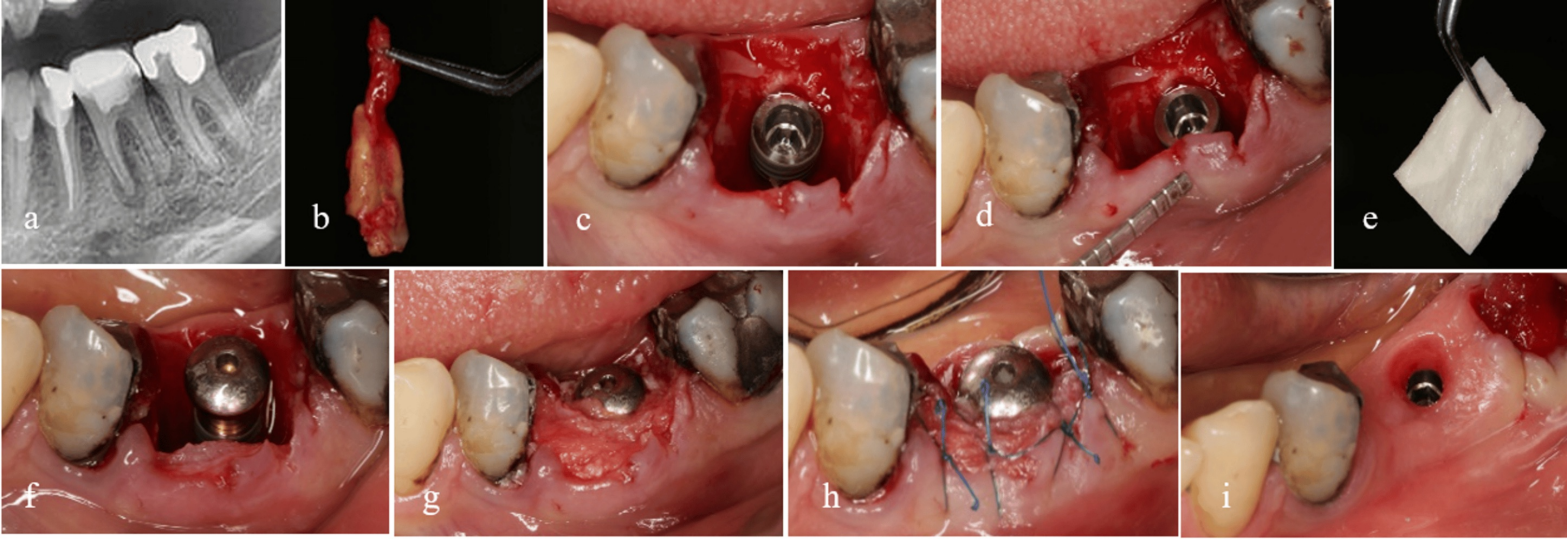
Type 4 of the biomechanical classification. Endodontic problems have caused the destruction of the buccal plate and a significant portion of the septal bone (a, b). Immediate implant placement was done using the lingual wall and the bone present in the apical recess, and adequate initial stability was obtained (c). In cases with the destruction of the buccal plate, the use of a barrier membrane is mandatory (d-f). Coronal flood was obtained with the help of healing abutment and membrane (g,h). The final image shows the running room and proper contour around the implant after 3 months (i).

In Type 4, where both variables are compromised, the implant placement necessitates careful attention to achieving PS and ensuring an effective coronal seal of the socket. This specific type of socket requires adherence to the PASS principles in GBR [17]. To address deficiencies in the buccal wall, it is feasible to position the membrane utilizing a tunneling technique from the coronal aspect beneath the soft tissue. Employing slow-resorption bone material can enhance resistance to minimize the risk of membrane collapse. Furthermore, a robust coronal seal plays a crucial role in enhancing the predictability of bone formation within the socket. According to the inherent capability of the socket to facilitate soft tissue development at its orifice [18], the use of a wide healing abutment can further support the establishment of an effective coronal seal. The presence of a blood clot near the socket orifice promotes epithelial proliferation, similar to its interaction with the connective tissue, thereby inhibiting epithelial penetration into the socket. Consequently, a natural coronal seal of the socket adjacent to a wide healing abutment can develop within a brief timeframe. In instances where PS is insufficient during implant placement, utilizing soft tissue grafts, such as a free gingival graft (FGG), can be an advantageous option for securing the healing abutment. Additionally, soft tissue grafting may be necessary to refine contouring in such cases. An illustrative example of the interplay of these variables is depicted in Figure 9. Furthermore, the application of thick fascia lata as a barrier membrane has proven beneficial in enhancing the surrounding soft tissue thickness in this particular scenario.

Decision-making diagram

**Figure 10:**
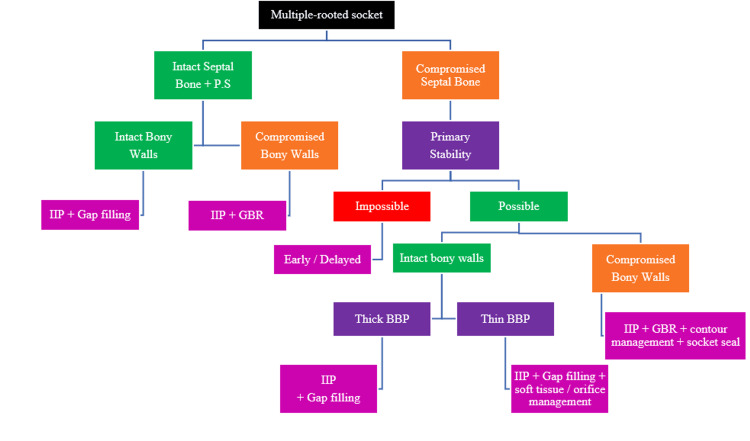
Decision-making diagram for biomechanical classification (Author's own creation). PS: Primary Stability, BBP: Buccal Bone Plate, GBR: Guided Bone Regeneration, IIP: Immediate Implant Placement

The decision-making diagram for IIP across various biomechanical classification types is presented in Figure 10.

## Conclusions

Numerous variables can influence the outcomes of immediate implant placement in multi-rooted sockets. Existing classifications are unable to interpret all variables concurrently. However, the biomechanical classification highlights the beneficial role of the septal bone in maintaining the blood supply to the buccal plate, thereby reducing the risk of its collapse. This classification facilitates effective socket management during IIP. A key requirement for IIP across various socket types is achieving PS. The BBT becomes particularly critical when the tenting role of the septal bone is compromised. Generally, the implementation of a barrier membrane is recommended in any buccal plate destruction.
